# Computational Modeling of Mediator Oxidation by Oxygen in an Amperometric Glucose Biosensor

**DOI:** 10.3390/s140202578

**Published:** 2014-02-07

**Authors:** Dainius Šimelevičius, Karolis Petrauskas, Romas Baronas, Razumienė Julija

**Affiliations:** 1 Faculty of Mathematics and Informatics, Vilnius University, Didlaukio 47, LT-08303 Vilnius, Lithuania; E-Mails: karolis.petrauskas@mif.vu.lt (K.P.); romas.baronas@mif.vu.lt (R.B.); 2 Institute of Biochemistry, Vilnius University, Mokslininku 12, LT-08662 Vilnius, Lithuania; E-Mail: julija.razumiene@bchi.vu.lt

**Keywords:** modeling, reaction-diffusion, biosensor, oxygen, aerobic, anaerobic

## Abstract

In this paper, an amperometric glucose biosensor is modeled numerically. The model is based on non-stationary reaction-diffusion type equations. The model consists of four layers. An enzyme layer lies directly on a working electrode surface. The enzyme layer is attached to an electrode by a polyvinyl alcohol (PVA) coated terylene membrane. This membrane is modeled as a PVA layer and a terylene layer, which have different diffusivities. The fourth layer of the model is the diffusion layer, which is modeled using the Nernst approach. The system of partial differential equations is solved numerically using the finite difference technique. The operation of the biosensor was analyzed computationally with special emphasis on the biosensor response sensitivity to oxygen when the experiment was carried out in aerobic conditions. Particularly, numerical experiments show that the overall biosensor response sensitivity to oxygen is insignificant. The simulation results qualitatively explain and confirm the experimentally observed biosensor behavior.

## 1. Introduction

A chemical sensor is a device that transforms chemical information, ranging from the concentration of a specific sample component to total composition analysis, into an analytically useful signal. Chemical sensors usually contain two basic components connected in series: a chemical (molecular) recognition system (receptor) and a physicochemical transducer. Biosensors are chemical sensors in which the recognition system utilizes a biochemical mechanism [1–3].

A biosensor recognition system is usually enzyme-based. Enzymes are known for their characteristic trait to catalyze only one specific chemical reaction, which provides biosensors with high specificity. Furthermore, enzymes are known to be very efficient catalysts, ensuring high biosensor sensitivity [4–8].

A physicochemical transducer may be developed by employing several different approaches. One of the most common approaches is amperometric. In this case, one of the products of chemical reactions occurring during the operation of a biosensor engages in an electrochemical reaction on the biosensor electrode surface. The measured current is usually proportional to the concentration of the analyte, which allows the determination of the analyte concentration using a preestablished calibration curve [2,5,9].

Carbon nanotubes are a popular electrode material among biosensor scientists. A lot of biosensors have been developed using carbon nanotube electrodes [10,11]. Some biosensors based on the direct electron transfer between an enzyme and a carbon nanotube electrode were designed [12,13]. However, it was demonstrated that it is possible to achieve direct electron transfer using a cheaper electrode material, modified carbon black [14,15]. In [15], a glucose biosensor employing the s-PQQ-glucose dehydrogenase (GDH) enzyme and the electrode based on carbon black was created. In this work, the glucose biosensor based on the same electrode and the same enzyme employing mediator N-methylphenazonium methyl sulfate was developed. To increase the stability and prolong the calibration curve of the biosensor, the enzyme was immobilized onto a polyvinyl alcohol (PVA) coated terylene film. Thus, the biosensor has the shape of a sandwich, where the permeable membrane entraps the enzyme layer [3,16]

The understanding of intrinsic biosensor mechanisms is important in designing and optimizing novel biosensors. In order to fully understand the processes taking place during biosensor operation, a mathematical model of a biosensor should be built [17,18]. Various mathematical models of biosensors have been built and successfully used so far [19–21]. Different sandwich-type (multilayer) biosensors have been also mathematically modeled [22–25]. Comprehensive reviews on the mathematical modeling of amperometric biosensors have been presented [26,27].

PQQ-dependent enzymes do not react with molecular oxygen [15]; thus, the biosensors presented in [15] do not require anaerobic conditions during operation. However, the biosensor presented in this paper uses a mediator, which does react with molecular oxygen [28]. The goal of this paper is to assess the extent of oxygen's influence on biosensor operation if it is used in aerobic conditions. A mathematical model of a glucose biosensor presented in this paper has been developed recently [29]. The model did not consider the oxidation of a mediator by molecular oxygen present in the bulk solution. The new model was created in order to model the influence of oxygen on the biosensor response.

The biosensor behavior was numerically analyzed at various values of input parameters of the model. The influence of the diffusion, as well as of the mediator's oxidation by oxygen on the biosensor response were thoroughly investigated.

## 2. Experimental

Aiming to design a biosensor electrode powder, carbon black RAVEN-Mobtained from Columbian Chemicals Co. (Atlanta, GA, USA) was mixed with a pasting liquid consisting of 10% polyvinyl dichloride in acetone and further was extruded, forming a tablet [30]. The tablet was sealed in a Teflon tube. The electrode was washed with bidistilled water and dried before use. As a biological recognition element, soluble PQQ-dependent glucose dehydrogenase (sPQQ-GDH) from *Acinetobacter calcoaceticus*, E.C.1.1.5.2 was used. The sPQQ-GDH was isolated and purified by the method reported in [31]. The enzyme was immobilized on individual flexible supports of 0.1% polyvinyl alcohol coated terylene.

The thickness of the terylene membrane was of 12μm. A thin layer of the PVA was formed on the terylene membrane. It was estimated that the thickness of this layer was about 1 μm.

All electrochemical measurements were performed using the electrochemical analyzer, PARSTAT 2273 (Princeton Applied Research, US), with a conventional three-electrode system containing the carbon paste electrode as a working electrode, a platinum wire as a counter electrode and an Ag/AgCl in saturated KCl as a reference electrode (all potential values presented in this paper are versus this reference electrode). The measurements were performed in potentiostatic conditions at *E* = 0.4V. Acetate buffer (50 mmol/L, pH = 6.0) was used as a default buffer. All measurements were carried out at an ambient room temperature (20 °C).

The initial experiments were conducted in both anaerobic and aerobic conditions. However, the difference in the signal between anaerobic and aerobic conditions was not observed. Thus, the rest of the experiments were conducted in aerobic conditions. The data used in this paper are for experiments conducted in aerobic conditions.

## 3. Mathematical Model

### 3.1. Reaction Scheme

We consider that the following chemical reactions take place during the operation of the biosensor [15,28,32,33]:

(1) $${\text{GDH}}_{\text{ox}}+\text{glucose}\overset{k_{1}}{\to }{\text{GDH}}_{\text{red}}+\text{gluconolactone}$$

(2) $${\text{GDH}}_{\text{red}}{+\text{PMS}}_{\text{ox}}\overset{k_{2}}{\to }{\text{GDH}}_{\text{ox}}{+\text{PMS}}_{\text{red}}$$

(3) $${\text{PMS}}_{\text{red}}+{\text{O}}_{2}\overset{k_{3}}{\to }{\text{PMS}}_{\text{ox}}{+\text{HO}}_{2}^{-}$$

(4) $${\text{PMS}}_{\text{red}}\to {\text{PMS}}_{\text{ox}}{+2e}^{-}$$

During the first chemical reaction, glucose dehydrogenase oxidizes glucose to gluconolactone. During the second chemical reaction, the reduced form of glucose dehydrogenase (GDH_red_) is oxidized by the mediator, N-methylphenazonium methyl sulfate (PMS), and regains its primary oxidized form (GDH_ox_). The third reaction is the oxidation reaction of the mediator by the oxygen that is present in the solution. During this reaction, the mediator is oxidized and regains its primary oxidized form. The fourth reaction is an electrochemical reaction that takes place on an electrode surface. During this reaction, the mediator is oxidized in the same way as in the third reaction.

Reactions (3) and (4) are competitive, as they both are dependent on the same reactant, PMS_red_. A high rate of Reaction (3) may reduce the concentration of PMS_red_ and, consequently, the rate of Reaction (4) and, thus, the electric current, which is the biosensor response.

For the sake of simplicity, further in this paper, we use an abstract notation of chemical species. As the purpose of the biosensor is the measurement of the glucose concentration, glucose is called the substrate and denoted as S; gluconolactone is called the product and denoted as P_1_; E_ox_ denotes GDH_ox_; E_red_ denotes GDH_red_; M_ox_ is PMS_ox_; and M_red_ is PMS_red_. P_2_ denotes the product of the third reaction: ${\text{HO}}_{2}^{-}$. Thus, the reaction schemes (1)–(4) transforms to:

(5) $${\text{E}}_{\text{ox}}+\text{S}\overset{k_{1}}{\to }{\text{E}}_{\text{red}}{+\text{P}}_{1}$$

(6) $${\text{E}}_{\text{red}}{+\text{M}}_{\text{ox}}\overset{k_{2}}{\to }{\text{E}}_{\text{ox}}{+\text{M}}_{\text{red}}$$

(7) $${\text{M}}_{\text{red}}{+\text{O}}_{2}\overset{{\text{k}}_{3}}{\to }{\text{M}}_{\text{ox}}{+\text{P}}_{2}$$

(8) $${\text{M}}_{\text{red}}\to {\text{M}}_{\text{ox}}+2e^{-}$$

### 3.2. Biosensor Principal Structure

The biosensor consists of three layers of different diffusivity of the species. The mathematical model should consider all these layers plus a diffusion layer, where concentrations of the substances differ from the ones in a bulk solution. In our mathematical model, we consider the Nernst model of a diffusion layer, which suggests that the diffusion front is stopped by the convection at a certain distance from the electrode. The profiles of concentrations inside a diffusion layer acquire linear shapes at a steady state. On the contrary, the semi-infinite model of the diffusion layer considers that the diffusion front may infinitely shift to the bulk of the solution. However, if the measurement time is not very short, it is indispensable to take into consideration the consequences of convection, as well [34–36].

**Figure 1.**
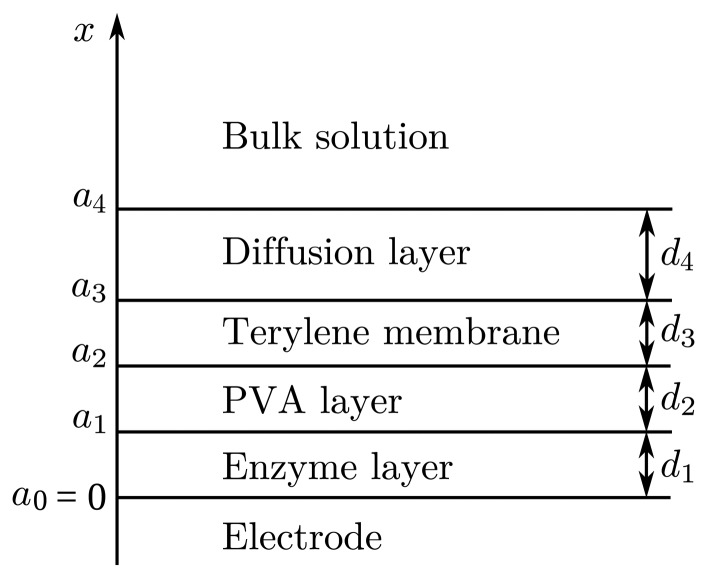
The principal structure of a biosensor. PVA, polyvinyl alcohol.

Let us define *d*_1_, *d*_2_, *d*_3_ and *d*_4_ as the thicknesses of the enzyme, PVA, terylene membrane and diffusion layers, respectively. We will also need values representing the distances between the electrode surface and the boundaries of the regions. Let *a*_1_, *a*_2_, *a*_3_ and *a*_4_ be the distances between the electrode surface and one of those boundaries, as shown in Figure 1. The thicknesses of all layers are specified in Table 1. Thicknesses d_1_ and d_4_ were not measured during the experiment. Their values were estimated by the model.

In the enzyme layer, biochemical and chemical reactions, as well as mass transport by diffusion take place. It is considered that the molecules of the enzyme are large enough and immobile; thus, diffusion does not influence the concentrations of both enzyme forms. Only the mass transport by diffusion and a chemical reaction (7) take place in the PVA layer, terylene membrane and diffusion layer, as enzyme molecules are absent in these three layers of the biosensor.

### 3.3. Governing Equations

The governing equations for a chemical reaction network can be formulated by the law of mass action [4,21]. Coupling reactions in the enzyme layer with the one-dimensional-in-space diffusion, described by Fick's second law, leads to the following equations of the reaction-diffusion type (0 < *x* < *a*_1_, *t* > 0):

(9) $$\frac{\partial e_{\text{ox}}}{\partial t}=-k_{1}e_{\text{ox}}s_{1}+k_{2}e_{\text{red}}m_{\text{ox},1}$$

(10) $$\frac{\partial e_{\text{red}}}{\partial t}=k_{1}e_{\text{ox}}s_{1}-k_{2}e_{\text{red}}m_{\text{ox},1}$$

(11) $$\frac{\partial s_{1}}{\partial t}=D_{\text{S},1}\frac{\partial^{2}s_{1}}{\partial x^{2}}-k_{1}e_{\text{ox}}s_{1}$$

(12) $$\frac{\partial m_{\text{ox},1}}{\partial t}=D_{{\text{M}}_{\text{ox}},1}\frac{\partial^{2}m_{\text{ox},1}}{\partial x^{2}}-k_{2}e_{\text{red}}m_{\text{ox},1}+k_{3}m_{\text{red},1}o_{1}$$

(13) $$\frac{\partial m_{\text{red},1}}{\partial t}=D_{{\text{M}}_{\text{red}},1}\frac{\partial^{2}m_{\text{red},1}}{\partial x^{2}}+k_{2}e_{\text{red}}m_{\text{ox},1}-k_{3}m_{\text{red},1}o_{1}$$

(14) $$\frac{\partial o_{1}}{\partial t}=D_{{\text{O}}_{2},1}\frac{\partial^{2}o_{1}}{\partial x^{2}}-k_{3}m_{\text{red},1}o_{1}$$

where *x* stands for space, *t* is time, *e*_ox_(*x, t*) and *e*_red_(*x, t*) correspond to concentrations of oxidized (E_ox_) and reduced (E_red_) enzyme, respectively; *s*_1_(*x, t*) is the concentration of the substrate in the enzyme layer; *m*_ox,1_(*x, t*) and *m*_red,1_(*x, t*) are the concentrations of the oxidized (M_ox_) and reduced (M_red_) forms of the mediator in the enzyme layer, *o*_1_ is the concentration of oxygen in the enzyme layer and *D*_S,1_, *D*_M_ox_,1_, *D*_M_red_,1_, *D*_O_2_,1_ are the diffusion coefficients of the corresponding substances defined by the subscript. In the definition of the concentrations and diffusion coefficients, here and later in this paper, the last numeric subscript label denotes the region of the model, particularly, 1 stands for the enzyme layer. The molecules of both enzyme forms, E_ox_ and E_red_, are considered as immobile, and therefore, there are no diffusion terms in the corresponding equations.

Products P1 and P2 do not act as reactants in any reaction, so their concentrations are not used in any further calculations. Therefore, Equations (9)-(14) contain no equations for product P1 and P2.

No enzyme molecules appear in other layers of the biosensor. Hence, only the mass transport by diffusion of species and the reaction (7) take place in other biosensor regions. The governing equations for these layers are represented as follows: (*a_i_*_−1_ < *x*< *a_i_*, *t* > 0, *i* = 2,3,4):

(15) $$\frac{\partial s_{i}}{\partial t}=D_{\text{S},i}\frac{\partial^{2}s_{i}}{\partial x^{2}}$$

(16) $$\frac{\partial m_{\text{ox},i}}{\partial t}=D_{{\text{M}}_{\text{ox}},i}\frac{\partial^{2}m_{\text{ox},i}}{\partial x^{2}}+k_{3}m_{\text{red},i}o_{i}$$

(17) $$\frac{\partial m_{\text{red},i}}{\partial t}=D_{{\text{M}}_{\text{red}},i}\frac{\partial^{2}m_{\text{red},i}}{\partial x^{2}}-k_{3}m_{\text{red},i}o_{i}$$

(18) $$\frac{\partial o_{i}}{\partial t}=D_{{\text{O}}_{2},i}\frac{\partial^{2}o_{i}}{\partial x^{2}}-k_{3}m_{\text{red},i}o_{i}$$

where *i* = 2 corresponds to the PVA layer, *i* = 3 corresponds to the terylene membrane layer and *i* = 4 corresponds to the diffusion layer.

### 3.4. Initial Conditions

Let *x* = *a*_0_ = 0 represents the electrode surface, while *x* = *a*_1_, *x* = *a*_2_, *x* = *a*_3_ and *x* = *a*_4_ represent the boundaries between the adjacent regions, as described in Section 3.2 and shown in Figure 1. The biosensor operation starts when the substrate and mediator appear in the bulk solution. This is used in the initial conditions (*t* = 0),

(19) $$\begin{array}{ccc} e_{\text{red}}(x,0)=0, & e_{\text{ox}}(x,0)=e_{0}, & 0<x<a_{1} \end{array}$$

(20) $$\begin{array}{ccc} s_{i}(x,0)=m_{\text{ox},i}(x,0)=m_{\text{red},i}(x,0)=0, & a_{i-1}\leq x\leq a_{i}, & i=1,2,3 \end{array}$$

(21) $$\begin{array}{cc} s_{4}(x,0)=m_{\text{ox},4}(x,0)=0, & a_{3}\leq x<a_{4} \end{array}$$

(22) $$\begin{array}{cc} m_{\text{red},4}(x,0)=0, & a_{3}\leq x\leq a_{4} \end{array}$$

(23) $$\begin{array}{cc} s_{4}(a_{4},0)=s_{0}, & m_{\text{ox},4}(a_{4},0)=m_{0} \end{array}$$

(24) $$\begin{array}{ccc} o_{i}(x,0)=o_{0}, & a_{i-1}\leq x\leq a_{i}, & i=1,2,3,4 \end{array}$$

where *e*_0_ stands for the total concentration of the enzyme in the enzyme layer (*e*_0_ = *e*_ox_(*x, t*) + *e*_red_(*x, t*), ∀*x*, *t* : *x* ∈ (0, *a*_1_), *t* > 0), *s*_0_ stands for the substrate concentration, *m*_0_ is the concentration of the oxidized form of the mediator and *o*_0_ is the concentration of the oxygen in the bulk solution.

### 3.5. Matching Conditions

On the boundary between two adjacent regions having different diffusivities, matching conditions have to be defined (*t* > 0, *i* = 1, 2, 3) [26,27],

(25) $${D_{\text{S},i}\frac{\partial s_{i}}{\partial x}|}_{x=a_{i}}{=D_{\text{S},i+1}\frac{\partial s_{i+1}}{\partial x}|}_{x=a_{i}},\;s_{i}(a_{i},t)=s_{i+1}(a_{i},t)$$

(26) $${D_{{\text{M}}_{\text{ox}},i}\frac{\partial m_{\text{ox},i}}{\partial x}|}_{x=a_{i}}{=D_{{\text{M}}_{\text{ox}},i+1}\frac{\partial m_{\text{ox},i+1}}{\partial x}|}_{x=a_{i}},\;m_{\text{ox},i}(a_{i},t)=m_{\text{ox},i+1}(a_{i},t)$$

(27) $${D_{{\text{M}}_{\text{red}},i}\frac{\partial m_{\text{red},i}}{\partial x}|}_{x=a_{i}}{=D_{{\text{M}}_{\text{ox}},i+1}\frac{\partial m_{\text{red},i+1}}{\partial x}|}_{x=a_{i}},\;m_{\text{red},i}(a_{i},t)=m_{\text{red},i+1}(a_{i},t)$$

(28) $${D_{{\text{O}}_{2},i}\frac{\partial o_{i}}{\partial x}|}_{x=a_{i}}{=D_{{\text{O}}_{2},i+1}\frac{\partial o_{i+1}}{\partial x}|}_{x=a_{i}},\;o_{i}(a_{i},t)=o_{i+1}(a_{i},t)$$

where *i* = 1 corresponds to the boundary between the enzyme layer and the PVA layer, *i* = 2 corresponds to the boundary between the PVA layer and the terylene membrane, whereas *i* = 3 corresponds to the boundary between the terylene membrane and the diffusion layer.

These conditions mean that fluxes of the species through one region are equal to the corresponding fluxes entering the surface of the neighboring region. Concentrations of species in one region *versus* the neighboring region are assumed to be equal.

### 3.6. Boundary Conditions

In the bulk solution, the concentrations of species remain constant (*t* > 0),

(29) $$s_{4}(a_{4},t)=s_{0}$$

(30) $$m_{\text{ox},4}(a_{4},t)=m_{0}$$

(31) $$m_{\text{red},4}(a_{4},t)=0$$

(32) $$o_{4}(a_{4},t)=o_{0}$$

The reduced mediator, M_red_, takes part in electrochemical Reaction (8) at the electrode surface (*x* = 0). The rate of this reaction is considered so high that the concentration of M_red_ at the electrode surface is permanently reduced to zero (*t* > 0),

(33) $$m_{\text{red},1}(0,t)=0$$

Since electrochemical Reaction (8) produces as much M_ox_ as it consumes M_red_, the flux of M_ox_ on the electrode surface is equal to the flux of M_red_, but in the opposite direction. This relation is expressed by the following boundary condition (*t* > 0):

(34) $${D_{{\text{M}}_{\text{red}},1}\frac{\partial m_{\text{red},1}}{\partial x}|}_{x=0}{=-D_{{\text{M}}_{\text{ox}},1}\frac{\partial m_{\text{ox},1}}{\partial x}|}_{x=0}$$

The substrate, S, and oxygen, O_2_, are electrode-inactive species; thus, their fluxes on the electrode surface are equal to zero (*t* > 0),

(35) $${D_{\text{S},1}\frac{\partial s_{1}}{\partial x}|}_{x=0}=0$$

(36) $${D_{{\text{O}}_{2},1}\frac{\partial o_{1}}{\partial x}|}_{x=0}=0$$

### 3.7. Biosensor Response

The measured current is usually assumed as the response of an amperometric biosensor in physical experiments. The biosensor current, *i*(*t*), at time *t* was expressed explicitly from Faraday's and Fick's laws,

(37) $${i(t)=An_{e}FD_{{\text{M}}_{\text{red}},1}\frac{\partial m_{\text{red},1}}{\partial x}|}_{x=0}$$

where *i*(*t*) is the faradaic current generated by electrochemical Reaction (8), *A* is the electrode surface, *n_e_* is the number of electrons involved in a charge transfer at the electrode surface and *F* is the Faraday constant, *F* = 96, 486 C/mol.

We assume that the system approaches a steady state as *t* →∞

(38) $$i_{\text{st}}=\underset{t\to \infty }{\lim}i(t)$$

where *i*_st_ is the the steady-state biosensor current.

Let us introduce *B*_O_2__, which shows the biosensor response sensitivity to oxygen,

(39) $$B_{{\text{O}}_{2}}=\frac{i_{st}(o_{0}=0)-i_{st}(o_{0}=c_{{\text{O}}_{2}})}{i_{st}(o_{0}=0)}$$

where *i_st_*(*o*_0_ = 0) is the biosensor response in anaerobic conditions, *i_st_*(*o*_0_ = *c*_O_2__) is the biosensor response in aerobic conditions and *c*_O_2__ = 2.53 × 10^−7^ mol/m^3^ is the oxygen concentration in water [40].

## 4. Numerical Simulation of Biosensor Action

### 4.1. Simulating the Biosensor Operation

The exact analytical solution for the problem (9)–(36) is not known. Therefore, the problem was solved numerically, using the finite difference technique [43,44]. An implicit finite difference scheme was built on a uniform discrete grid with 50 points in the space direction for each modeled layer corresponding to a certain time moment. The simulator has been programmed by the authors in the C++ programming language [45].

In the numerical simulation, the biosensor response time was assumed as the time when the change of the biosensor current remains very small during a relatively long term. A special dimensionless decay rate, *ε*, was used,

(40) $$t_{\text{r}}=\underset{i(t)>0}{\min}\left\{t:\frac{t}{i(t)}\left|\frac{\text{d}i(t)}{\text{d}t}\right|<\varepsilon \right\},\;i(t_{r})\approx i_{\text{st}}$$

where *t*_r_ is the biosensor response time. The decay rate value *ε* = 10^−2^ was used in the calculations.

### 4.2. Model Validation

**Figure 2.**
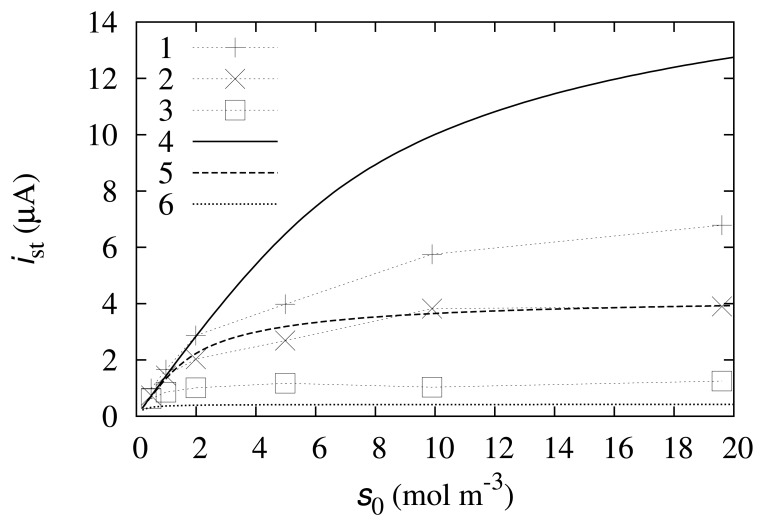
Experimental (1, 2, 3) and computational (4, 5, 6) calibration curves at different concentrations of the mediator (*m*_0_): 0.2mol/m^3^ (1, 4), 0.05mol/m^3^ (2, 5), 0.005 mol/m^3^ (3, 6).

**Figure 3.**
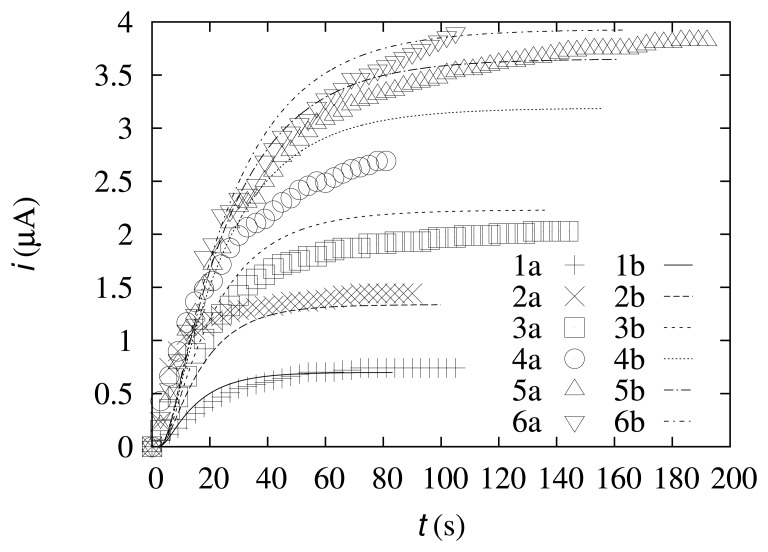
The dynamics of the biosensor current at different values of the substrate concentration, *s*_0_: 0.49 mol/m^3^ (1), 0.99 mol/m^3^ (2), 1.99 mol/m^3^ (3), 4.98 mol/m^3^ (4), 9.9 mol/m^3^ (5), 19.6 mol/m^3^ (6); (**a**) experiment; (**b**) simulation.

The numerical solution of the model (9)–(36) was compared with the experimental data. The results are depicted in Figures 2 and 3.

As one can observe from Figure 2, the simulated calibration curve at *m*_0_ = 0.05 mol/m^3^ fits the experimental data well. At higher and lower mediator concentrations, the modeled data do not fit the experimental results so well. This may be explained by some processes that are not accounted for in the mathematical model. These processes may include reverse reactions, the instability of the compounds or enzyme degradation. Furthermore, some reaction rate constants, *k*_1_, *k*_2_ or *k*_3_, may exhibit dependence on the mediator concentration.

The dynamics of biosensor responses during the experiments was compared with the results of the computational experiments. The data are depicted in Figure 3.

As one can observe from Figure 3, the dynamics of the simulated biosensor response is in good agreement with the experimental data. At lower concentrations of the substrate (*s*_0_ = 0.49 mol/m^3^ and *s*_0_ = 0.99 mol/m^3^), the current difference at the steady state is 6.1% and 7.5%, respectively. At the intermediate concentrations (*s*_0_ = 1.99 mol/m^3^ and *s*_0_ = 4.98 mol/m^3^), the current difference at the steady state is slightly higher (9.7% and 18%, respectively). At higher concentrations of the substrate (*s*_0_ = 9.9 mol/m^3^ and *s*_0_ = 19.6 mol/m^3^), the differences in the steady-state current are 4.6% and 0.58%, respectively. These results show that the simulated data more accurately coincide with the experimental data at relatively low and high substrate concentrations.

### 4.3. Concentration Profiles at the Steady State

**Figure 4.**
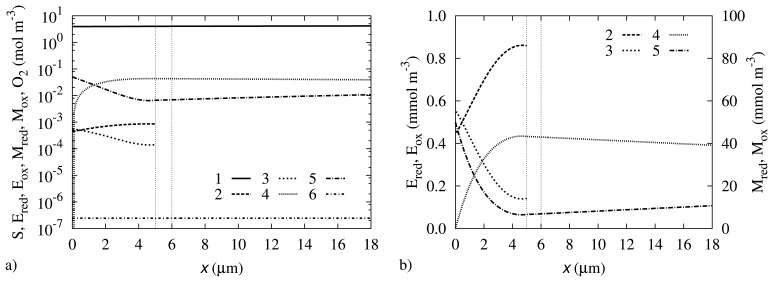
The concentration profiles of S (1), E_red_ (2), E_ox_ (3), M_red_ (4), M_ox_ (5) and O_2_ (6) at the steady state. Vertical axes correspond to the concentration of species, horizontal axes correspond to the distance from an electrode surface. The vertical lines mark the boundaries of the biosensor layers. Concentrations in graph (a) are provided in logarithmic scale. In graph (b) concentrations of four species are depicted on a linear scale.

Concentration profiles may be a useful source of information about the processes that take place during the biosensor operation. The biosensor action was simulated at the parameter values as specified in Table 1. The concentration profiles at the steady state are depicted in Figure 4.

One can observe in Figure 4 that the concentration of oxygen is almost the same through all the investigated region. The oxygen concentration at the electrode surface is only 3.6% lower than in the bulk solution. This indicates that the rate of Reaction (7) is comparatively slow in comparison with the diffusion of this gas.

The curve representing the concentration of M_red_ shows that the concentration at the electrode surface is reduced to zero, due to the fast electrochemical Reaction (8). The same electrochemical reaction causes an increase in the concentration of M_ox_ at the electrode surface, where it has practically the same concentration as in the bulk. In the middle region, the concentration of M_red_ is higher than the concentration of M_ox_.

The concentration of the substrate at the electrode surface is equal to 3.93 mol/m^3^, which is 21% less than in the bulk solution. This significant reduction in the concentration shows that the rate of Reaction (5) is comparatively high in comparison with the diffusion of the substrate from the bulk solution.

One can observe that the concentrations of E_red_ and E_ox_ correlate with the concentrations of M_red_ and M_ox_, respectively. While in the middle regions, the E_red_ concentration is higher than E_ox_, it is the opposite at the electrode surface. The concentration shift at the electrode surface may be explained by the fact that E_red_ is the reactant in Reaction (6), the rate of which is significantly increased by the increase in the M_ox_ concentration, which is the second reactant of Reaction (6).

### 4.4. The Biosensor Response Sensitivity to Oxygen

In order to assess the biosensor response sensitivity to oxygen, the biosensor operation was simulated in both aerobic and anaerobic conditions. *B*_O_2__ was used as a measure of the sensitivity.

**Figure 5.**
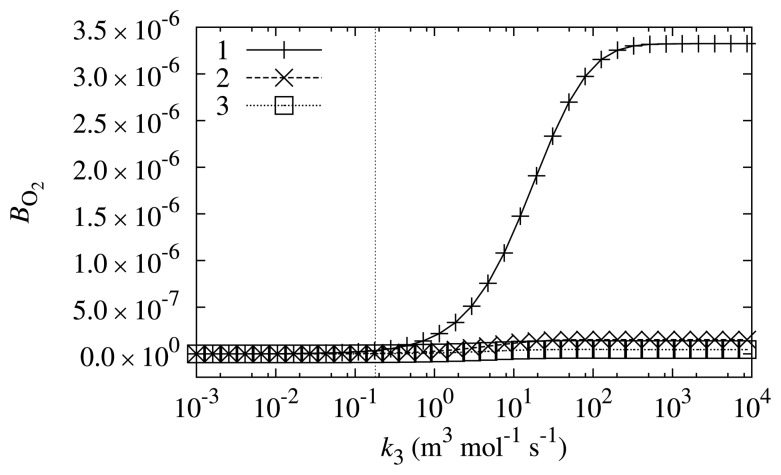
The biosensor response sensitivity, *B*_O_2__, to oxygen dependence on reaction rate constant of Reaction (7), *k*_3_, at different values of substrate concentration *s*_0_: 0.498 mol/m^3^ (1), 4.98 mol/m^3^ (2) and 49.8 mol/m^3^ (3). The vertical line marks the reaction rate constant, *k*_3_, corresponding to N-methylphenazonium methyl sulfate (PMS).

In order to investigate what properties a compound should have to be a suitable mediator, the biosensor response sensitivity, *B*_O_2__, dependence on *k*_3_ was investigated at different values of the substrate concentration, *s*_0_. The other biosensor parameters were equal to the values indicated in Table 1. The results are depicted in Figure 5.

The values of *B*_O_2__ depicted in Figure 5 indicate that the design of the biosensor is very successful. The simulated biosensor response sensitivity to oxygen shows that the oxygen influence is about 6–7 orders of magnitude smaller than the biosensor response itself. From the viewpoint of biosensor sensitivity to oxygen, the PMS is a very successful mediator, as the value of *B*_O_2__ is very low at the *k*_3_ value corresponding to PMS. These results are in accord with physical experiments, which did not observe a difference in readings while conducting the same experiment in both anaerobic and aerobic conditions.

Even though the biosensor response sensitivity to oxygen is small, it is dependent on the value of *k*_3_ and substrate concentration *s*_0_. Two main tendencies may be observed from the curves depicted in Figure 5: the oxygen has a bigger influence when *k*_3_ is bigger and when the substrate concentration, *s*_0_, is smaller. Bigger values of reaction rate constant *k*_3_ results in higher rates of Reaction (7). Therefore, this directly influences the value of *B*_O_2__. However, if the concentration of the substrate is high, the change in *k*_3_ only slightly alters the values of *B*_O_2__. At lower substrate concentrations, the shift towards higher biosensor response sensitivity to oxygen is more significant if *k*_3_ increases.

**Figure 6.**
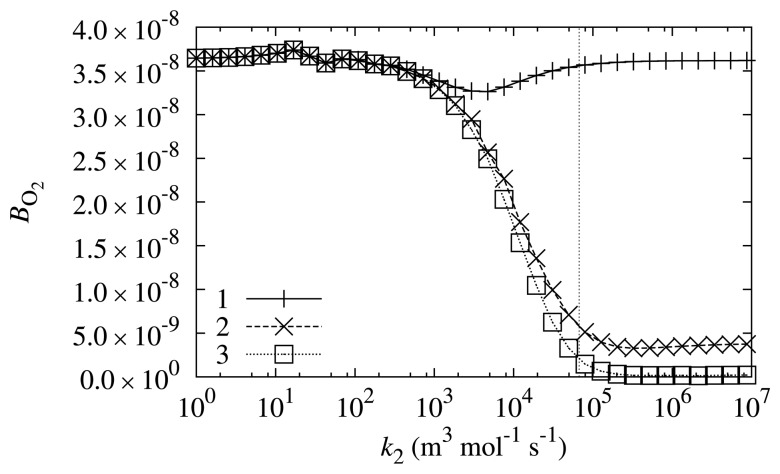
The biosensor response sensitivity, *B*_O_2__, to oxygen dependence on reaction rate constant of Reaction (6), *k*_2_, at different values of substrate concentration *s*_0_: 0.498 mol/m^3^ (1), 4.98 mol/m^3^ (2) and 49.8 mol/m^3^ (3). The vertical line marks the reaction rate constant, *k*_2_, corresponding to PMS.

The rate of Reaction (6) is also dependent on the properties of a mediator; particularly, it is directly dependent on the reaction rate constant, *k*_2_. The biosensor response sensitivity, *B*_O_2__, to oxygen dependence on *k*_2_ was investigated at different values of the substrate concentration, *s*_0_. The results are depicted in Figure 6.

As is evident from Figure 6, the biosensor response sensitivity to oxygen is even less dependent on the reaction rate constant, *k*_2_. The main tendency that may be observed from Figure 6 is that biosensor sensitivity *B*_O_2__ is lower at higher values of reaction rate constant *k*_2_. The sensitivity of the biosensor to oxygen at *k*_2_ value corresponding to PMS is one of the lowest in the range of investigated sensitivities.

However, besides the main tendency, one may observe that at lower substrate concentrations (*s*_0_ = 0.498 mol/m^3^), the dependency is non-monotonous. The biosensor response sensitivity to oxygen is the lowest when the value of reaction rate constant *k*_2_ is equal to approximately 5 × 10^3^ m^3^ mol^−1^ s^−1^. At lower and higher values of *k*_2_, the biosensor shows a higher sensitivity to oxygen.

**Figure 7.**
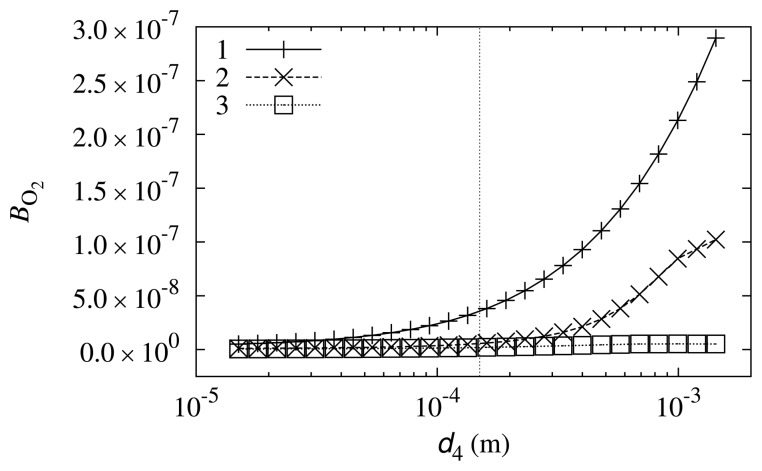
The biosensor response sensitivity, *B*_O_2__, to oxygen dependence on the thickness, *d*_4_, of the diffusion layer at different values of the substrate concentration, *s*_0_:0.498 mol/m^3^ (1), 4.98 mol/m^3^ (2) and 49.8 mol/m^3^ (3). The vertical line marks the estimated diffusion layer thickness during the physical experiment.

The impact of the diffusion layer thickness, *d*_4_, on the biosensor response sensitivity to oxygen was also investigated. Simulation results are depicted in Figure 7.

The curves in Figure 7 show that the impact of diffusion layer thickness on the biosensor sensitivity to oxygen is small. However, the tendency that a thicker diffusion layer results in a higher oxygen influence may be observed. This may be explained by the fact that the diffusion coefficient of oxygen is the highest among the compounds that are reactants in reaction scheme (5)–(8) (see Table 1). The thicker the diffusion layer, the faster that oxygen reaches the biosensor compared with the other compounds.

## 5. Conclusions

The mathematical model (9)–(36) describes the processes that take place during the biosensor operation sufficiently well. The model may be used as a tool for assessing the need to conduct the measuring experiment with a biosensor in anaerobic conditions. The model may be also used to investigate other properties and behaviors of a biosensor.

The comparison of experimental and simulated results showed that the model most accurately describes the biosensor operation at moderate mediator concentrations (*m*_0_ = 0.05 mol/m^3^). At lower and higher concentrations of the mediator, the accuracy is lower.

The investigation of simulated concentration profiles at the steady state showed that the consumption of oxygen is very low. This shows that the rate of reaction consuming molecular oxygen is low and should not significantly influence the biosensor response when an experiment is carried out in aerobic conditions.

The investigation of the biosensor response at different parameter values showed that overall, the biosensor response sensitivity to oxygen is very small. The change in response in anaerobic conditions is six to seven orders of magnitude smaller than the biosensor response itself. It was shown that the mediator was very successfully chosen from the viewpoint of biosensor response sensitivity to oxygen.

It was also shown that a thicker diffusion layer slightly increases the biosensor response sensitivity to oxygen. This is consistent with the fact that the diffusion coefficient of oxygen is the highest among the compounds participating in chemical reactions during biosensor operation.

Simulated results and conclusions inferred from them should be used with caution, as the biosensor model poorly fits the experimental data with some mediator concentrations. This may indicate that some processes are not accounted for in the biosensor model or that some reaction rate constants used in the model exhibit dependence on the mediator concentration.

## Figures and Tables

**Table 1.** The parameters of the model.

**Parameter**	**Value**	**Reference**
*d*_1_	5 × 10^−6^ m	estimation
*d*_2_	1 × 10^−6^ m	experiment
*d*_3_	1.2 × 10^−5^ m	experiment
*d*_4_	1.5 × 10^−4^ m	estimation
*D*_S,1_, *D*_M_ox_,1_,*D*_M_red_,1_	1.5 × 10^−10^ m^2^/s	estimation
*D*_S,2_,*D*_M_ox_,2_,*D*_M_red_,2_	4.2 × 10^−10^ m^2^/s	estimation
*D*_S,3_,*D*_M_ox_,3_,*D*_M_red_,3_	3.75 × 10^−10^ m^2^/s	estimation
*D*_S,4_	6.77 × 10^−10^ m^2^/s	[37]
*D*_M_ox_,4_, *D*_M_red_,4_	4.57 × 10^−10^ m^2^/s	[38]
*D*_O_2_,1_,*D*_O_2_,2_,*D*_O_2_,3_	1.970 × 10^−9^ m^2^/s	estimation
*D*_O_2_,4_	1.970 × 10^−9^ m^2^/s	[39]
*e*_0_	1 × 10^−3^ mol/m^3^	estimation
*m*_0_	5 × 10^−2^ mol/m^3^	experiment
*s*_0_	4.98 mol/m^3^	experiment
*o*_0_	2.53 × 10^−7^ mol/m^3^	[40]
*k*_1_	8.1 × 10^2^ m^3^ mol^−1^ s^−1^	[41]
*k*_2_	6.7 × 10^4^ m^3^ mol^−1^ s^−1^	[42]
*k*_3_	1.8 × 10^−1^ m^3^ mol^−1^ s^−1^	[28]
*n*_e_	2	[33]
*A*	4.5 × 10^−6^ m^2^	experiment
